# More Than Morningness: The Effect of Circadian Rhythm Amplitude and Stability on Resilience, Coping, and Sleep Duration

**DOI:** 10.3389/fpsyg.2021.782349

**Published:** 2021-11-16

**Authors:** Lee Di Milia, Simon Folkard

**Affiliations:** ^1^School of Business and Law, Central Queensland University, Rockhampton, QLD, Australia; ^2^Department of Psychology, Swansea University, Swansea, United Kingdom

**Keywords:** amplitude, flexibility, languid, circadian, phase, resilience

## Abstract

Self-report tools that measure circadian rhythms have focused primarily on phase. We add to the sparse literature on assessing amplitude and stability. We randomly recruited 1,163 participants who completed several measures. The correlation between the LV scale (amplitude) and FR scale (stability) was −0.12 (*p* < 0.01). As expected, amplitude was negatively associated with phase (*r* = −0.64, *p* < 0.01) while stability showed a weak link with phase (*r* = 0.07, *p* < 0.05). Structural equation modeling suggested a close model-fit of the factor structure in the sample (RMSEA = 0.033). The LV scale explained 22% of the variance, while the FR scale explained 23%. Scale reliability was satisfactory for the LV scale (0.68) and good for the FR scale (0.73). Participants with low amplitude or flexible rhythms reported significantly better resilience, coping, and required less daily sleep. We constructed a composite circadian categorical variable to combine the best attributes from the LV and FR scales; participants with *both* low amplitude and flexible rhythms, reported significantly better resilience, coping, and less sleep need. We found rhythm amplitude decreased with age, while stability remained constant.

## Introduction

Interest in developing self-report survey tools to assess the human circadian rhythm appears vibrant given recent developments in this field. Circadian rhythms can be understood in terms of three key characteristics; (a) phase, the timing of the rhythms’ peak and trough across the 24 h; (b) amplitude, the difference between the rhythms peak and trough, and (c) stability refers to the predictive constancy of the amplitude. The literature has primarily focused on assessing phase (Adan et al., 2012). In contrast, the literature on rhythm amplitude and stability is sparse. The first attempt to measure these characteristics was the Circadian Type Questionnaire (CTQ, Folkard et al., 1979), followed by the revised Circadian Type Inventory (rCTI; Di Milia et al., 2004). The rCTI has been validated and used extensively to assess tolerance to working in non-standard working arrangements such as shift work (Flo et al., 2012; Chen et al., 2020). However, the last decade has seen several new scales purporting to assess amplitude. These include the Chronotype Questionnaire (Oginska et al., 2017), the Circadian Amplitude and Phase Scale (Di Milia et al., 2011), and the “distinctiveness” scale from the Morningness-Eveningess Stability Scale (Randler et al., 2016). These scales are yet to report detailed relationships with tolerance to shift work. In this article we assess the utility of the two-factor rCTI beyond studies of shift workers and examine its relationship with resilience, coping and sleep duration. In doing so, the rCTI may be used in a broader range of studies to uncover the relationship between circadian rhythm changes and well-being.

The CTQ (Folkard et al., 1979) was initially developed to assess phase, amplitude, and stability with a primary focus to predict adjustment to shift work. Theoretically, the CTQ was built on the premise that “better adjustment might be shown by people with; (a) low amplitude rhythms, and (b) flexible or non-stable rhythms” (p. 80). Assessment of rhythm amplitude is operationalized via the *languid-vigor* scale with more vigorous types thought to have a lower amplitude and therefore, better adjustment to night work, or shift-work tolerance. The stability of the circadian rhythm is assessed with the *flexible-rigid* scale, with more flexible types showing less stability and therefore, better able to cope with night work. Vigorous and flexible types are considered to better deal with sleep loss or delaying sleep.

The factor structure of the rCTI was developed with a student sample (Di Milia et al., 2004) and replicated using a working sample (Di Milia et al., 2005). Recently, Pallesen et al. (2021) confirmed the factor structure and its stability over an 8-year period in a nursing sample. The results from several cross-sectional and longitudinal studies support Folkard et al.’s (1979) propositions. Di Milia et al. (2005) reported significant differences in alertness by time of day between languid (high amplitude) and vigorous (low amplitude) types. Vigorous types also reported less sleep inertia on waking and needing less sleep. Significant differences in alertness across the day were also found between flexible and rigid types.

In cross-sectional studies, Flo et al. (2012) investigated shift-work disorder in 2,000 nurses and found symptoms were negatively linked with flexibility (OR = 0.92) but positively linked with languidity (OR = 1.10). In a study focused on “severe” shift-work disorder, languidity (OR = 1.28) was as a significant predictor Saksvik-Lehouillier et al. (2012a), and (Di Milia et al., 2013) reported languidity was negatively related to shift-work tolerance, while flexibility was positively related.

We found three longitudinal studies that support the importance of low amplitude and flexible rhythms for well-being. In a study of 706 intern nurses languidity (OR = 1.70), but not flexibility (OR = 0.70) predicted shift work disorder at a 6-month follow up (Chen et al., 2020). In a sample of nurses working a three-shift schedule, Saksvik-Lehouillier et al. (2012b) reported that flexibility was negatively associated with anxiety 1 year later, while languidity was associated with greater sleepiness and fatigue.

We have three main goals for this study. First, we test via confirmatory factor analysis the posited factor structure for the rCTI (Di Milia et al., 2005). While the model was replicated in student (Di Milia et al., 2004) and shift-work samples (Pallesen et al., 2021), we are unaware of replication studies based on random samples. Replicating the structure in a random population suggests the potential for the rCTI to be applied in a wider number of settings.

Second, we extend our knowledge concerning the distribution of rhythm amplitude and stability. We assess the relationship between these rhythm parameters and age, gender, resilience, coping, and sleep duration. While the phase advance relationship with age is well known (Fischer et al., 2017), there is less data suggesting the association between age, rhythm amplitude and stability in large samples. Given the age-related decay of the circadian system, we would expect a reduction in amplitude (Duffy et al., 2015). Similarly, eveningness is associated with impaired affect (Cox and Olatunji, 2021), depression (Hasler et al., 2010), resilience and optimism (Antúnez et al., 2015). However, there is no literature to suggest the relationship between these variables with amplitude and stability.

Third, we revisit Smith et al. (1989) proposition that a “weighted combination” of rhythm characteristics may be a better predictor of positive adjustment compared with relying on a single rhythm indicator. We identified one study that created a composite construct by forming a languid-rigid group and a flexible-vigorous group (Di Milia et al., 2005). The results indicated the flexible-vigorous group was significantly more alert across the day. We hypothesize that people with vigorous *and* flexible (i.e., low amplitude and non-rigid rhythms) rhythms will be associated with better outcomes than a languid-rigid group (high amplitude and rigid rhythms).

## Materials and Methods

### Participants and Procedure

As part of a larger study, trained telephone interviewers used random digit dialing to contact 2,323 residents in three regional Australian cities. Calles were made in the evening hours and each person was contacted up to three times across consecutive days before being discarded. Participants were at least 18 years of age and in paid employment.

Participants were told the purpose of the study was to assess the link between work and well-being, that participation was voluntary, and they could cease participation at any time. Participants provided verbal consent before completing the interview. The study protocol was approved by the University’s Human Ethics Research Committee (H11/09-149).

### Instruments

Participants provided their details to several demographic variables, such as age, gender, their work schedule, and work experience. In addition, they completed the following scales.

#### Revised Circadian Type Inventory

The *languid-vigor* (LV) scale contains 6-items and is purported to measure rhythm amplitude. High scores suggest greater languidity and difficulty with nightwork. A sample item is, “*Do you find it difficult to “wake-up” properly if you are awoken at an unusual time?”* The *flexible-rigid* (FR) scale has 5-items to assess rhythm stability. Higher scores indicate flexibility and better adjustment to nightwork. A sample item is, “*Do you enjoy working at unusual times of day or night?”*

#### Morning Affect

The Morning Affect (MA) scale (Di Milia and Bohle, 2009) is derived from the Composite Scale of Morningness (Smith et al., 1989) and contains 4-items that assess morningness preference only. A sample item is, *“During the first half hour after having woken in the morning, how tired do you feel?”* Higher scores indicate greater morningness.

#### Brief Resilience Scale

The Brief Resilience Scale (BRS) (Smith et al., 2009) is a 6-item scale that examines the ability to rebound from stressful events. A sample item is, *“I usually come through hard times with little trouble.”* Higher scores suggest greater resilience.

#### Brief Resilience Coping Scale

The Brief Resilience Coping Scale (BRCS) (Sinclair and Wallston, 2004) is a 4-item scale measuring adaptive coping to stress. A sample item is, *“I actively look for ways to replace the losses I encounter in my life.”* Higher scores suggest better coping.

#### Sleep Duration

We posed a single self-reported question to assess sleep duration; “*How many hours of actual sleep do you usually get during a normal 24-h period? (This may be different than the number of hours you spend in bed).”*

### Data Analysis Strategy

Prior to conducting confirmatory factor analysis (CFA, AMOS, V26) we examined the items for missing data. Little’s MCAR test (χ^2^ = 138.80, df = 154, *p* < 0.81) suggested missing values (2.8%) were completely at random and we used the EM procedure (SPSS V26) to replace missing data. Large samples are not appropriate for CFA (Gatignon, 2010) and we conducted the CFA based on a 20% random sample (*n* = 253) using maximum likelihood estimation.

To assess the distribution of LV and FR by age we created five age categories: ≤ 30, > 30–40, > 40–50, > 50–60, ≥ 60.

To detect the effect of low or high amplitude, and flexible or stable rhythms, we used the 30th and 70th percentile to establish four groups. The 30th percentile from the LV scale was used to establish a “vigor” group (*n* = 217), and the 70th percentile defined the languid group (*n* = 239). Similarly, the 30th percentile from the FR scale defined the rigid group (*n* = 208) while the 70th percentile defined the flexible group (*n* = 248).

We used the 30th and 70th percentile to create two composite groups. The LR group consisted of participants that scored ≥ 70th percentile on the LV scale and ≤ 30th percentile on the FR scale. The FV group is derived from participants that scored ≤ 30th percentile from LV and ≥ 70th percentile from the FR scale.

## Results

The sample (*n* = 1163, 50% response rate) consisted of 623 females (54%) and 540 males. Mean age was 45.30 years (*SD* = 11.20). Most were full-time workers (70.40%) and the balance worked part-time. The mean weekly working hours were 38.83 h (*SD* = 14.59) and approximately 78% were cohabitating. Approximately 23% of the sample worked rotating shifts across the 24-h period.

The LV scale explained 22%, and the FR scale explained 23% of the variance (KMO = 0.77, *p* < 0.001). Model fit for the posited two-factor structure suggested an excellent fit (χ^2^ = 195.41; *p* > 0.05). Several incremental fit indices indicated a close-fitting model to the sample (Hu and Bentler, 1999); comparative fit index (0.97), the Tucker-Lewis index (0.97), the RMSEA (0.033), and the SRMR (0.058). Standardized regression weights for items ranged from 0.36 to 0.62 for the LV scale and from 0.36 to 0.79 on the FR scale.

Descriptive statistics, correlation matrix and Cronbach alpha for the variables can be found in Table 1. Skew and kurtosis for scale scores were within the acceptable range (−1 to 1). All scales showed satisfactory to good levels of reliability.

**TABLE 1:** Descriptive statistics, correlations, and Cronbach alpha (diagonal) for scale scores.

	Mean	*SD*	1	2	3	4	5	6
1. Age	45.27	11.23						
2. Languid/Vigor	14.70	5.53	−0.25**	0.68				
3. Flexible/Rigid	14.10	5.41	−0.06*	−0.12**	0.73			
4. Morningness	12.22	2.75	0.25**	−0.64**	0.07*	0.82		
5. Resilience	3.54	0.69	0.03	−0.35**	0.17**	0.33**	0.80	
6. Coping	14.59	3.14	0.04	−0.10**	0.09**	0.10**	0.27**	0.68
7. Sleep duration/day	6.92	1.06	−0.15**	0.15**	−0.18**	−0.07*	0.00	0.03

*p < 0.05, **p < 0.001.

Pearson correlation between the LV and FR scale was −0.12 (*p* < 0.001) and after controlling for morningness, the correlation was −0.11 (*p* < 0.001). Morningness and LV were negatively linked (−0.64, *p* < 0.001), while the association between FR and morningness was weak (0.07, *p* < 0.05). Cronbach alpha for the LV scale was 0.68. The item-total correlations ranged from 0.30–0.50. Cronbach alpha for the FR scale was 0.73 and the item-total correlations ranged from 0.32 to 0.62.

A multivariate GLM with age as a covariate identified a significant gender difference [Pillai’s trace, *F*(2, 1086) 23.99, *p* < 0.001]. Females reported a higher mean for LV (*M* = 15.20, *SD* = 5.60; Males *M* = 14.35, *SD* = 5.42) while males, were higher on FR (*M* = 15.24, *SD* = 5.35; Females *M* = 13.08, *SD* = 5.28). The effect size was small (η^2^ = 0.04) based on Eta squared.

**FIGURE 1:**
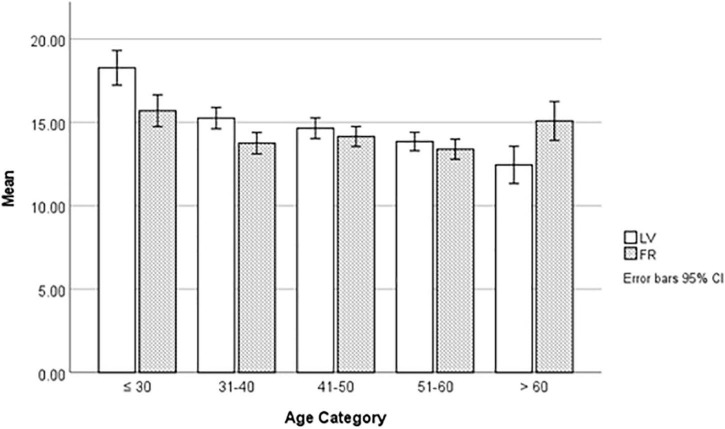
Distribution of languid-vigor and flexible-rigid types by age categories.

A one-way ANOVA by age categories identified significant differences in LV and FR (see Figure 1). Bonferroni *post hoc* tests indicated significant mean reductions in LV across age categories. The effect size for the mean differences was medium (η^2^ = 0.07) based on Eta squared. Mean scores for FR showed a small decline across age categories and the effect size was small (η^2^ = 0.02). However, significant differences were only detected between the ≤ 30 and the 31–40 age groups, and the ≤ 30 age and 51–60 age group.

A multivariate GLM identified significant mean differences between languid and vigor types across each dependent variable [Pillai’s trace, *F*(5, 448) 64.67, *p* < 0.001]. Languid types were less resilient, less able to cope, and needed more sleep (see Table 2). The effect size was large for morningness (η^2^ = 0.39), approaching large for resilience (η^2^ = 0.12) and small for the remaining variables. Significant differences were also obtained between flexible and rigid types [Pillai’s trace, *F*(5, 448) 10.79, *p* < 0.001]. Mean scores suggested rigid types were less resilient, less able to cope, and needed more sleep than flexible types. The effect size for these differences were small to medium. Mean scores were not significantly different between flexible and rigid types on morningness. The results also indicated a significant interaction between the two groups [Pillai’s trace, *F*(5, 448) 2.84, *p* < 0.05] with a small effect size (η^2^ = 0.01).

**TABLE 2:** Mean scores and effect size for languid-vigor (*n* = 456) and Flexible-Rigid (*n* = 456) types by dependent variables.

	Languid	Vigor	*p*	η^2^	Flexible	Rigid	*p*	η^2^
Morningness	10.03 (2.86)*	13.95 (1.76)	0.001	0.39	12.19 (3.13)	11.62 (3.03)	ns	0.00
Resilience	3.25 (0.78)	3.79 (0.61)	0.001	0.12	3.69 (0.65)	3.29 (0.82)	0.001	0.01
Coping	14.10 (3.20)	15.04 (3.19)	0.001	0.02	14.94 (3.18)	14.08 (3.23)	0.001	0.06
Sleep duration	7.11 (1.08)	6.62 (1.08)	0.001	0.04	6.65 (1.12)	7.15 (1.03)	0.001	0.05

*Standard deviation; η^2^, eta squared.

Regarding the composite variable, the FV group comprised of 65 females and 75 males (*n* = 140) and the LR group had 96 females and 39 males. A χ^2^-test indicated these groups were significantly different (17.25, df, 1,275, *p* < 0.001). The FV group reported a mean age of 47.87 years (*SD* = 10.45) compared to 44.07 (*SD* = 9.35) for the LR group (*t* = 3.16, df, 2,271, *p* < 0.001).

A χ^2^-test indicated these groups differed across age categories (χ^2^ = 16.68, df = 4,275, *p* < 0.001). In percentage terms, LR declined with age while FV increased with age. Within age groups, LR was more common < 30 years of age but FV was more common > 60 years of age (see Table 3).

**TABLE 3:** Percentage* of languid-rigid (*n* = 135) and flexible-vigor (*n* = 140) types by age categories.

	<30 years	31–40 years	41–50 years	51–60 years	>60 years
Languid-rigid	67	57	49	50	10
Flexible-vigor	33	43	51	50	90

*Rounded to whole number.

We ran a multivariate GLM to test the LR and FV groups for differences against the dependent variables. These mans can be found in Table 4. Pillai’s trace was significant [*F*(5, 251) 51.46, *p* < 0.001] and the effect size approached medium (η^2^ = 0.05). The means for the FV group indicated they were significantly (*p* < 0.001) more morning oriented, resilient, coped better, and required less sleep. The effect size for these mean comparisons was large for morningness, resilience and sleep duration.

**TABLE 4:** Mean scores and effect size for Languid-rigid (*n* = 124) and flexible-vigor (*n* = 133) types by dependent variables.

	Languid-rigid	Flexible-vigor	*p*	η^2^
Morningness	10.18 (2.83)*	14.00 (1.76)	0.01	0.42
Resilience	3.05 (0.86)	3.88 (0.61)	0.01	0.24
Coping	13.87 (3.28)	15.45 (3.17)	0.01	0.06
Sleep duration	7.24 (1.05)	6.37 (1.06)	0.01	0.15

*Standard deviation; η^2^, eta squared.

## Discussion

The literature on self-reported circadian rhythm is dominated by studies on rhythm phase (Adan et al., 2012). In contrast, we focused on assessing rhythm amplitude and stability, and in doing so, we add to the literature in several ways.

Theoretically, circadian rhythm phase (morningness), amplitude and stability are considered distinct constructs. The correlation between amplitude and stability (−0.12) suggested they are not related. Furthermore, their correlation with morningness also indicated the three constructs are unique. However, there was a weak correlation between morningness and the FR scale (0.07, *p* < 0.05) indicating a small overlap between these constructs. However, overall, these data suggest the independence of the three rhythm parameters.

The psychometric properties of the rCTI (Di Milia et al., 2005) appear to be sound. We replicated the posited two-factor structure, and the results of the CFA showed an excellent model fit in a random sample. This is an important finding because it suggests the rCTI can be used in a broader range of studies on psycho-social well-being. The factor structure was recently replicated in a large nursing sample and was found to be stable over an 8-year period (Pallesen et al., 2021).

Scale reliability for the LV and FR scales produced mixed results. A consistent finding is that Cronbach alpha for the FR scale tends to be about 0.75 and we found a similar value. However, we obtained a Cronbach alpha pf 0.68 for the LV scale and the literature suggests values less than 0.70 may be problematic (Hinkin, 1998). We inspected the item-total correlations for the LV items and found each item correlated with the scale total ≥ 0.30, suggesting these were medium associations (Streiner et al., 2014). Deleting items would not have increased the reliability coefficient. Other studies, however, have reported higher scale coefficients for the LV scale (Saksvik-Lehouillier et al., 2012b; Pallesen et al., 2021).

Mean scores suggested males have a more flexible rhythm than females and this finding aligns with other studies (Marcoen et al., 2015). Based on prior studies (Di Milia et al., 2004) males with a flexible rhythm reported needing less daily sleep. In a supplementary analysis we found males indicated they required 0.14 h less sleep per day (*p* < 0.05*;* Cohen’s *d* = 0.14). Other studies have reported flexibility is also associated with higher levels of alertness across the day and night (Di Milia et al., 2004; Marcoen et al., 2015).

The literature suggests aging is associated with a reduction in rhythm amplitude in several physiological markers. For example, Czeisler et al. (1992) reported that the amplitude of the temperature rhythm fell in older men by some 20–40%. Other studies have reported amplitude reductions in melatonin (Zhao et al., 2002), cortisol (Van Cauter et al., 1996) and blood pressure (Hermida et al., 2013) in older participants. Our results also demonstrated amplitude decreased with age. Figure 1 suggests the largest mean difference can be observed about 30 years of age. The findings are unlikely to be explained by the 10-year categories we created, since the association can be seen in the correlation matrix. Furthermore, Pallesen et al. (2021) also identified a reduction in the mean LV score over an 8-year period but found, amplitude and flexibility scores at baseline were positively associated with their respective follow-up scores. Figure 1 suggests a U-shaped relationship between age and the stability of the rhythm. However, we caution the number of participants in the > 60 age group were small. Excluding this age group also suggests stability decreases with age but the changes are less marked than the decline in amplitude.

The explanation for the age-related changes to the efficacy of the circadian system is attributed to the weakening of the supra-chiasmatic nucleus (SCN) located in the hypothalamus (Cornelissen and Otsuka, 2017; Hood and Amir, 2017) such that it becomes less responsive to environmental cues such as light (Roenneberg et al., 2003). One line of reasoning is these changes occur via the degradation of the visual system. The thickening of the lens reduces the amount of light entering the SCN resulting in molecular changes that disrupt the firing of circadian rhythms (Duffy et al., 2015; Hood and Amir, 2017). The SCN is considered as the principal molecular clock that regulates behavior and physiology (Weaver, 1998).

One of our goals was to explore the role of amplitude and stability with respect to individual differences. The languid (high amplitude) and rigid (non-flexible) participants were significantly less resilient than the respective vigorous and flexible participants. Similar to Saksvik-Lehouillier et al. (2015) we found a small but significant correlation indicating languid types had poorer coping styles, while flexible types coped better. We posit some possibilities that explain the relationship between circadian rhythm, resilience, and coping.

Developments in technology have opened new perspectives in understanding resilience. Adopting a biopsychosocial model suggests a complex interaction between our genetic makeup, gene variants, the environment and neurobiology (Feder et al., 2019). Neuroimaging studies are providing insights into the neural indicators of psychological traits. For example, Forero et al. (2020) reported a link between a functional polymorphism in the monoamine oxidase and coping. It may be possible that the breakdown of the SCN also drives reduced resilience and coping indirectly via changes in sleep duration. Age related changes in reduced sleep duration, advanced timing of sleep and sleep disruption is a robust finding into middle adulthood (Li et al., 2018); a period that shows changes in rhythm amplitude and stability (see Figure 1). Sleep loss was reported as a key factor in lower levels of resilience in a study of 55,000 service personnel (Seeling et al., 2016). Sleep loss also leads to neuro-behavioral and molecular changes that may explain how individuals respond to stressors (Goel et al., 2013).

The final goal of our study was to respond to Smith et al.’s (1989) call to explore whether a combination of rhythm parameters may have better diagnostic value. The results indicated that people with both vigorous and flexible rhythms (low amplitude and non-rigid) were significantly more resilient, coped better required less daily sleep. Another interesting finding was that the vigorous and flexible group tended to be more morning oriented. This suggests the possibility that a weighted combination of rhythm amplitude, it’s stability and morningness may provide additional diagnostic value. It is important to highlight that the composite group we constructed existed at a categorical, not scale level. Whether it is possible to measure and combine different rhythm characteristics onto a single scale is a challenge for future studies.

The distribution of languid-rigid and flexible-vigorous types showed an inverse relationship (Table 3). While the percentage of participants in the languid-rigid declined with advancing age, the percentage of participants in the flexible-vigor group increased. However, we caution there were few cases in the > 60 age group. It is not clear also whether this finding is due to changes in the circadian rhythm or age-related changes in lifestyle such that sleep is not restricted by work schedule and/or domestic demands.

The main strength of our study is we drew upon a large random population sample. This feature suggests we avoided the limitations of small samples, ones that are occupation specific, contain gender bias or age restriction; limitations that are prevalent in the literature. In addition, we achieved a response rate of 50% which aligns with Baruch’s (1999) guidelines that samples within 60 ± 20% do not require further consideration. A third strength is that we used the 30th and 70th percentiles to polarize the differences between the groups.

These strengths must be considered along with some limitations. The study employed a cross-sectional design, and this prohibits the possibility of making causal inferences between the variables. All data were self-reported raising the possibility of bias. Another limitation is we used a single item to assess sleep duration and future studies should obtain accurate information regarding sleep onset and offset. However, the use of some single items is common in large scale sleep (Seeling et al., 2016) and chronotype studies (Antúnez et al., 2015) to reduce the burden compliance for participants. There is also evidence suggesting single item measures can be reliable indicators (Ahlstrom et al., 2010).

Overall, this study provided new data on the utility of the rCTI (Di Milia et al., 2005). In replicating the factor structure in a random sample, we argue the measure is useful beyond shift work samples and can be used in other settings. We identified age-related changes in rhythm amplitude and stability, and found associations between these constructs, resilience, coping and sleep duration. Finally, we add further evidence that a composite circadian rhythm indicator can be used as an indicator of adjustment. We encourage future studies to employ better research designs to examine the relationship between amplitude, stability, and individual differences.

## Data Availability Statement

The raw data supporting the conclusions of this article will be made available by the authors, without undue reservation.

## Ethics Statement

The studies involving human participants were reviewed and approved by the Human Research Ethics Committee, Central Queensland University (H11/09-149). The patients/participants provided their written informed consent to participate in this study.

## Author Contributions

LDM designed the study, conducted the data analyses, and drafted the manuscript. SF provided feedback and cowrote the final submission. Both authors contributed to the article and approved the submitted version.

## Conflict of Interest

The authors declare that the research was conducted in the absence of any commercial or financial relationships that could be construed as a potential conflict of interest.

## Publisher’s Note

All claims expressed in this article are solely those of the authors and do not necessarily represent those of their affiliated organizations, or those of the publisher, the editors and the reviewers. Any product that may be evaluated in this article, or claim that may be made by its manufacturer, is not guaranteed or endorsed by the publisher.
